# The socioeconomic determinants of sustainable residential water consumption in Athens: empirical results from a micro-econometric analysis

**DOI:** 10.1007/s43621-021-00047-6

**Published:** 2021-10-20

**Authors:** Ioannis Kostakis

**Affiliations:** 1 Department of Economics and Sustainable Development, Harokopio University, Athens, Greece; 2 Department of Business Administration, Hellenic Open University, Patras, Greece

**Keywords:** Residential water demand, Water sustainability, Price elasticity, Income elasticity, Greece, D10, Q25

## Abstract

This paper provides empirical evidence on the effects of socioeconomic characteristics on residential water consumption. The case of Athens is taken as an example for the empirical investigation, using data from the 2019 Household Budget Survey. Employing ordinary, two- and three-stage least squares, seemingly unrelated regression equations and simultaneous quantile specifications, we found that residential water demand is highly price inelastic. Furthermore, empirical results show that water consumption is positively related to household age while more educated households and unemployed persons seem to follow more environmentally friendly behaviour with respect to water demand. Income, gender, house ownership and population density seem to insignificantly affect residential water demand. Our empirical findings might have important national and regional policy implications in the design of sustainable water demand management.

## Introduction

Water demand management is of paramount importance for the sustainability of the water system [1–4]. As the availability of water is expected to decrease in the future [5], policy design tools to guarantee its sustainable use play important role [6]. Regarding residential water, it is known that there are several factors that may affect its use such as own-price, income, household size, age, education level, climatic conditions and even legislation. However, water demand management is not an easy task as the main policy-relevant variable, water price, cannot be increased without issues for several reasons. First, an increase in water price will lead to a penalization on several household budget balances resulting in a lower utility, especially among low-income households. A smoother block rate structure can work as an alternative attractive option to encourage water conservation [7]. Second, based on the majority of the empirical studies [8–10], residential demand is highly inelastic^1^ cancelling out the effect of a real water price increase on water conservation. In addition, pricing policy is mainly regulated by local and government councils, and so a higher water price is a politically sensitive issue for decision-makers [1].

Regarding Greece, water management appears to be more problematic due to the severe water supply scarcity that is being forecast in the forthcoming years [11]. In the largest cities of the country (Athens and Thessaloniki), companies own and operate the treatment plants, while water supply is managed by municipal companies in the rest of the cities [12, 13]. As far as water consumption is concerned, Greece belongs to the group of European Union countries with a high level of household water use per capita^2^ [13]. Concerning price, the increasing block prices system is used. Especially in Athens, that is the city of interest in our analysis, the block prices are as follows: (a) €0.35 per cubic meter for the first 60.8 cubic meters [0–60.8 m^3^], (b) €0.64/m^3^ for the second block [60.8–243.3 m^3^] (c) €1.83/m^3^ for the third block [243.3–328.5 m^3^] (d) €2.56/m^3^ for the fourth block [328.5–425.8 m^3^] and (e) €3.20/m^3^ for the fifth block [> 425.8 m^3^].

In the empirical literature, there is a substantial body of published works that estimate residential water demand as a function of price, income, sociodemographic and household characteristics, and weather conditions [5, 8, 10, 14–18]. Regarding price elasticity, the majority of the empirical studies support that residential demand for water is highly inelastic (*see inter* alia Arbues et al. [9]; Martinez-Espiñeira [19]; Schleich and Hillenbrand [20] ;Yudhistira et al. [21]; Hung et al. [22]) but it can also differ between low-income and high-income households [23], between seasons [9] or because of different methodological approaches that are followed [24]. The general low (in absolute value) elasticity is expected as water represents a small share of total household expenditures without good substitutes. Similarly, income elasticity is positive but in general on the low side of estimates [20, 22, 25], confirming that water is a normal good. This finding is also expected as higher income is associated with better living standards and so higher water demand. Wealthier households might also move to bigger houses having more needs for appliances, gardens or swimming pools.

Concerning the non-economic factors, several variables seem to affect residential water demand. For instance, literature indicates that age distribution within the household affects residential water use. Regarding age, empirical results are mixed as there is some evidence which shows that older people, ceteris paribus, consume less water than younger people as they have less washing and hygiene needs [19, 24, 26] while others [20] find the converse, namely that as people get older they consume more water since they spend more time at home [27]. Household size is presented as another important determinant of residential water demand [15, 28]. Dwelling characteristics—mainly the size, the age of the house or the area of residence—might also affect water consumption [24, 29]. Education seems to positively related to water conservation attitude [17, 20]. Finally, climatic conditions (temperature, rainfall levels, solar radiation, etc.), are also considered to be significant factors of water demand [18–20, 30].

In our case, the purpose of this study is the investigation of residential water demand, taking as an example, the case of Athens (Greece) in 2019. Our paper contributes to the relevant literature in many respects. First, despite the large body of empirical literature on water demand, the estimates on this subject are still rare in Greece [11, 13, 31–33]. Second, it employs a number of static and quantile regression models also taking into account the reverse causality between water price and water consumption. Note that the existing empirical studies explore the behaviour of residential water demand mostly using panel data specifications. The findings might be a useful input for the design, assessment and forecasting of water management policy. They may also allow for the specification of efficient policy measures with respect to residential water demand. Finally, our empirical results might be useful for the investigation of other countries facing similar challenges. The rest of the paper is structured as follows. Section 2 reports the methodology applied and data used. Section 3 presents the main empirical results while Sect. 4 discusses the main findings and provides some final policy remarks.

## Data and models

### Data

We use individual household data derived from the annual Household Budget Surveys of the Hellenic Statistical Authority (ELSTAT, 2020) that allows us to capture household level effects. The final sample includes 2041 households from Athens for 2019.

### Models

First, we employ an ordinary least squares model as a benchmark estimation for our analysis. Thereafter, taking into account the endogeneity issue, the empirical study considers a cross section regression model based on a system of two equations describing the interconnections between residential water demand and price of water which are both endogenous to the system [34]. Thus, the empirical water demand model is represented by the following specification:

1 $${Ln}{\text{wdpc}}_{{\text{i}}} =\upbeta {Ln} {\text{wp}}_{{\text{i}}} +\upgamma {Ln} {\text{inc}}_{{\text{i}}} +\uptheta {\text{x}}_{{\text{i}}} +\upvarepsilon _{{\text{i}}} ,\quad {\text{i}} = 1,2, \ldots ,{\text{N}},$$

where the dependent variable ${Ln} {\text{wdpc}}_{{\text{i}}}$ of residential demand model is defined as the natural logarithm of residential water demand per capita ${\text{i}}$ at period ${\text{t}}$, $\upbeta$, $\upgamma$ and $\uptheta$ are the vectors of associated coefficients. Based on the traditional demand function, the main independent variables include ${Ln} {\text{nwp}}_{{\text{i}}}$ which is the natural logarithm of real price of household water consumption^3^ and ${Ln} {\text{inc}}_{{\text{i}}}$ which is the natural logarithm of annual real household disposable income; ${\text{x}}_{{\text{i}}}$ is a vector of other exogenous explanatory control variables such as the household sociodemographic characteristics, given by the education level, household structure with respect to age, gender, population density, etc. and $\upvarepsilon _{{\text{i}}}$ represents the disturbance term.

Residential water consumption in physical terms (cubic meters m^3^) is calculated by dividing the total nominal expenditure on water by the euro/m^3^ average price of water (calculated endogenously). In the empirical analysis we used the average block price per household. Nominal disposable income and prices are deflated by the national harmonized commodity price index base year of 2015. The descriptive statistics of the variables of interest are summarized in Table 1.

**Table 1:** Descriptive statistics

Variables	Mean	St. Dev.	Min	Max
Water consumption per capita (annual cubic meters)	72.41	40.03	8.68	353.20
Real average marginal block water price^a^ (Euro/m^3^)	0.49	0.09	0.35	1.35
Household disposable income (Euro per capita/year)	17,482	10,199	3,762	193,596
Number of unemployed members	0.97	0.97	0	5
Household size (number of members)	2.24	1.12	1	5
Number of kids under 15 years old	0.29	0.65	0	4
Number of elderly members over 65 years old	0.62	0.77	0	3
Tabulation				
Gender (male)	66.7%			
Higher educated households (university degree)	36.5%			
Population density (urban)	65.8%			
House ownership (tenants)	21.0%			

^a^Without taxes and levies

The second equation—price equation—is specified analogously as follows:

2 $${Ln} {\text{wp}}_{{\text{i}}} =\upbeta {Ln} {\text{wdpc}}_{{\text{i}}} +\upgamma {Ln} {\text{inc}}_{{\text{i}}} +\uptheta {\text{k}}_{{\text{i}}} +\upvarepsilon _{{\text{i}}} ,\quad {\text{i}} = 1,2, \ldots ,{\text{N,}}$$

where ${\text{k}}$ represents a group of exogenous variables that were included into our model [34]. Combining Eqs. (1) and (2), a reciprocal relationship is established between the residential water demand and water price variables which creates an endogeneity problem due to simultaneity. The water price variable is endogenous in the demand equation and the water demand variable is endogenous in the residential water price equation. To overcome this problem, these two equations are estimated jointly as a unique model by the two-(2SLS) and three-stage least squares (3SLS) approach respectively.^4^

Thereafter, to investigate whether the model coefficients might vary for different levels of water consumption within households, we also employed simultaneous bootstrapping quantile regressions (SBQR). In general, quantile regression specification allows flexibility in the estimated coefficients enabling us to obtain a range of conditional quantile functions, while it can remain robust to outliers. Thus, to account for possible heterogeneity among households, quantile regression analysis is employed as follows:

3 $${\text{Q}}_{{{\text{d}},{\text{i}}}} \left( {{\text{y}}_{{\text{i}}} |{\text{x}}_{{\text{i}}} } \right) = {\text{a}}\left( {\text{d}} \right) + {\text{x}}_{{\text{i}}}^{{\text{D}}} {\text{a}}_{{\text{x}}} \left( {\text{d}} \right) + {\text{F}}_{{{\text{e}},{\text{i}}}}^{ - 1} \left( {\text{d}} \right),$$

where ${\text{Q}}$ is the annual residential water consumption, ${\text{d}}$ is the quantile in the distribution of household water consumption and can take values between zero and unity; ${\text{x}}$ denotes the vector of independent variables and ${\text{a}}_{{\text{x}}} \left( {\text{d}} \right)$ is the varying effect of the explanatory variables. ${\text{F}}_{{{\text{e}},{\text{i}}}}^{ - 1} \left( {\text{d}} \right)$ represents the inverse of the cumulative distribution function of ${\text{e}}_{{\text{i}}}$. We followed simultaneous quantile regression since it produces quantile regression estimates for several values of quantiles simultaneously, allowing for differences between coefficients for different quantiles to be tested. Also, structural quantile function^5^ defined by Chernozhukov and Hansen [35] that uses the exogenous variables as instrumental variables in our model was employed giving very similar results.

## Empirical results

Table 2 presents the empirical results from the ordinary least squares, seemingly unrelated regression equations, the two- and three-stage least squares and the simultaneous quantile regression specifications.

**Table 2:** Empirical results based on OLS, SURE, 2SLS 3SLS and SBQR models, 2019

Variables	OLS	2SLS	3SLS	q = 0.25	q = 0.50	q = 0.75
Lnprice (natural log of price)	− 0.280*** (0.015)	− 0.119*** (0.006)	− 0.118*** (0.018)	− 0.148*** (0.010)	− 0.151*** (0.003)	− 0.138*** (0.004)
Lnincome (natural log of income)	− 0.004 (0.005)	0.000 (0.005)	0.001 (0.005)	0.000 (0.001)	0.001 (0.001)	0.001 (0.001)
Elderly (> 65 y.o.)	0.007*** (0.002)	0.006*** (0.002)	0.004** (0.002)	0.002** (0.001)	0.000 (0.000)	− 0.008** (0.000)
Kids (< 15 y.o.)	− 0.000 (0.004)	− 0.002 (0.004)	− 0.004 (0.004)	− 0.000 (0.001)	− 0.000 (0.000)	− 0.001 (0.001)
Gender (male)	0.001 (0.004)	− 0.005 (0.004)	− 0.004 (0.004)	− 0.000 (0.001)	− 0.001 (0.000)	− 0.002*** (0.001)
Education (University)	− 0.004 (0.004)	− 0.008** (0.003)	− 0.008** (0.004)	− 0.001 (0.001)	− 0.001** (0.000)	− 0.001*** (0.000)
Pop. density (urban)	− 0.002 (0.004)	0.000 (0.004)	0.000 (0.004)	− 0.000 (0.001)	− 0.0002 (0.000)	− 0.000 (0.001)
Occupation St. (unemployed)	0.013*** (0.003)	− 0.006* (0.003)	− 0.006* (0.003)	− 0.001* (0.000)	− 0.001*** (0.000)	− 0.002*** (0.000)
Housing type (tenant)	− 0.006 (0.004)	− 0.001 (0.005)	− 0.001 (0.005)	0.001 (0.001)	0.001 (0.001)	0.001 (0.001)
Constant term	0.216*** (0.050)	0.468*** (0.053)	0.473*** (0.053)	0.344*** (0.017)	0.329*** (0.006)	0.352*** (0.009)
CI [95%]	− 0.25/− 0.31	− 0.08/− 0.15	− 0.07/− 0.14	− 0.13/− 0.17	− 0.15/− 0.16	− 0.13/− 0.15

Structural equation model that uses as instruments of endogenous price variable the rest of covariates gives similar price elasticities with the simultaneous quantile regression estimators presented in Table 1. Thus, price elasticity equals − 0.163*** for q = 0.25, − 0.160*** for q = 0.50 and − 0.169*** for q = 0.75

No of obs: 1776. Standard errors in parentheses

CI [95%]: confidence intervals for water price elasticity; y.o.: years old

*p < 0.1

**p < 0.05

***p < 0.01

As shown in Table 2, residential water demand is found to be price inelastic and on the low side of literature [20, 24, 36, 37]. In particular, we found that if water price increases by 10%, the quantity demanded decreases by around 1 percentage point.^6^ Even observing confidence intervals (95%), price elasticity ranges from − 0.07 to − 0.34 across all models confirming its low elasticity. From a policy implications perspective, pricing reform’s effectiveness depends on the price elasticity of demand. This mechanism is also more effective when price elasticity is large. This means that in the case of Greece, average marginal price reforms might have a small effect in reducing water demand. It is also interesting that price elasticity remains a bit higher but stable across quantiles. In other words, price effect seems to be very similar between low intensity and high intensity water consumers in Athens.

Income elasticity of residential water demand is found to be highly insignificant so that a change in income level does not have any positive or negative effect on water demand. This finding, even if differs from several previous empirical studies [9, 11, 20], coincides with results found in the literature, where income elasticity can be an insignificant factor for residential water consumption. For instance, the European Commission provided some evidence that water is a normal good for most EU-28 countries, meaning that residential water use tends to increase with household income, but this is not the case for a group of countries including Greece [13]. This result can be also expected since water is an essential good with very low substitutability for all households [38].

Concerning household socioeconomic characteristics, we obtain evidence that education level could play a significant role on residential water demand management. A higher education level has a decreasing impact on household water use per capita. This finding is expected as households with higher educational level are more sensitive to environmental issues [39] and so decrease their residential consumption of water in their homes. However, education might be an insignificant factor on per capita water demand for low intensity consumers.

Regarding age structure of the households, we found that a higher ratio of elderly members within households significantly affects per capita consumption of residential water. In general, the empirical results of impact of the age on household water use per capita are mixed. On one hand, an aged population has different needs and so may consume less water. On the other hand, since elderly members spend more time at home they can consume larger quantities of water [5, 40]. Also, older people might use less modern and efficient appliances for washing needs and so consume more water. In our case, we found that a higher ratio of older members within household is associated with higher household water consumption per capita [19]. This also may indicate that younger households might use water more efficiently in their homes than elderly households. It should need to include this result in the design of demand-side policymaking. Occupation status (unemployed members) seems also to affect water demand. More specifically, we found that unemployed members seem to follow water conservation behaviour compared to employed members. Finally, concerning population density (urban or rural) of living, gender and house ownership type (tenants) we obtain small or no evidence for any impact on water consumption levels.

## Conclusions, limitations and policy implications

This paper provides evidence on the behaviour of residential water consumption in Greece using a cross section dataset. Our empirical investigation relies on the use of disaggregated household data of the independent annual Household Budget Survey conducted in 2019 by ELSTAT. The econometric investigation is carried out using static ordinary least squares, seemingly unrelated regression equations, two- and three-stage least squares and simultaneous quantile regression function models. The study might be useful input for the design and assessment of residential water management policies in other regions or countries sharing similar experiences.

The results show that residential water demand is highly price inelastic confirming many previous empirical studies. The value of the own-price elasticity is around − 0.13 on average across all estimated models.^7^ The level of education is a significant factor in the determination of household water consumption leading to water conservation behaviour. Also, we found that water demand is affected only marginally by household disposable income (elasticity equals − 0.000) confirming a European Commission technical report in the case of Greece. Household age and occupation status also seem to be important determinants of residential water demand, while the role of gender, area of living and house ownership are mainly insignificant factors. Regarding the quantile regression models, results confirm the low estimated price elasticity across households.

Empirical results may provide useful insights for future residential water management and policy design. In particular, the insignificant value of income elasticity of water demand (almost zero) shows that the expected future rise in incomes in Greece after the prolonged economic and Covid-19 recessions will not play a key role for residential water consumption. Regarding price reforms, the expected low water demand price elasticity makes the price reform mechanisms less efficient. For instance, an increase of 10% in water price will lead to only around 1–1.5% drop in residential water demand. The result is that large price increases are required to observe residential water conservation through the price mechanism.

However, other measures can stimulate water conservation. According to our results, more educated and younger households seem to follow a more environmentally friendly behaviour with respect to water consumption. Thus, policy measures focused on the behaviour of consumers including (i) the systematic dissemination of information to households, showing the efficient use of water in homes, (ii) the launching of educational programs making consumers more sensitive to residential water saving and (iii) campaigns for water-saving technologies or public awareness campaigns would have multiple benefits on the sustainability of residential water demand. These measures can smooth out water consumption resulting in a more efficient and wiser use of water within households.

Nevertheless, we should mention the limitations of the present study. Since Greece is a highly heterogenous country with respect to water consumption due to its specific regional and climatic conditions, a regional aspect of the household water consumption should be taken into consideration in the future research. Also, we only have annual aggregated information. However, possible seasonal data could give different elasticities for different seasons. Also, more micro datasets over time can give us the opportunity to employ more sophisticated models and have more robust results for water demand function under a micro-econometric aspect.

## Data Availability

The datasets generated during and/or analysed during the current study are available from the corresponding author on reasonable request.

## Footnotes

1 For some exceptions see, for instance, Kotagama et al. [41].

2 This result is also confirmed in our analysis in which household water use per capita is around 72 m^3^ per inhabitant in 2019.

3 In general, there is great debate about the appropriate price variable: average or marginal price. See for example, Martins and Fortunato [42].

4 This 3SLS approach takes place in 3 steps. Firstly, we regress the endogenous variables in relation to all exogenous variables taking the fitted values of this (reduced form of the system); secondly, we use the estimated fitted values of the endogenous regressor found in the first step, and retain the residuals (two-stage least squares); thirdly, we apply a generalized least squares (GLS) estimator on the joint form of the system using the residuals found in the second step to define the variance–covariance matrix of the error terms. We have gains in efficiency if we estimate the equations of the system jointly while controlling for endogeneity of regressors as we take into account the error-correlation between both equations.

5 ivqreg2 that was written by J. A. F. Machado and J. M. C. Santos Silva was used for this kind of analysis.

6 Without taking into account reserve causality, the price elasticity seems to be upward biased Reynaud et al. [43] while when addressing endogeneity, results are similar to [25] general findings.

7 Except OLS that does not take into account the endogeneity issue.
